# Buzzing boundaries: tiny caterpillars vibrate to defend leaf tip territories

**DOI:** 10.1242/jeb.249796

**Published:** 2025-04-01

**Authors:** Sarah M. Matheson, Leonardo M. Turchen, Emilie Mauduit, Jayne E. Yack

**Affiliations:** Department of Biology, Carleton University, 1125 Colonel By Drive, Ottawa, ON, Canada K1S 5B6

**Keywords:** Larva, Biotremology, Defence, Drepanidae, Lepidoptera, Communication

## Abstract

Territorial displays include some of the most elaborate behaviours in the animal kingdom. In this study, we investigated the territorial behaviour and vibratory signalling of neonate warty birch caterpillars (*Falcaria bilineata*; Lepidoptera: Drepanidae), which reside solitarily on birch leaves and defend the leaf tip. Upon hatching, these tiny caterpillars – no larger than 2 mm – seek out and establish a small solitary territory (∼1 cm wide) at the leaf tip, where they lay silk mats, feed and advertise their presence by producing multicomponent vibratory signals – buzz scrapes and drums. When a conspecific neonate (intruder) is introduced to a leaf occupied by a resident, the resident increases its signalling rate up to four times that when undisturbed, and even more – up to 14 times – if the intruder enters the territory. Intruders rarely manage to take over the resident's defended space, with most confrontations (71%) ending in the resident maintaining control. Residents signal significantly more than intruders at all stages of the contest. If physical contact occurs, residents flee by dropping from the leaf by a silk thread. This results in territorial contests that involve no physical aggression, relying entirely on vibratory communication. These vibratory displays most likely function to establish spacing between conspecifics on a tree branch, but these complex signals may also function to exclude other members of the vibratory community by mimicking something dangerous, such as a jumping spider.

## Teaser

**Highlighted Article:** Tiny warty birch caterpillars defend leaf tip territories by producing complex vibratory signals. Signals function as vibratory fences to exclude intruders, and leaf tips may offer escape routes from enemies.

## INTRODUCTION

Territorial behaviour is widespread throughout the animal kingdom and has been studied extensively in vertebrates and invertebrates (Baker, 1983; Bradbury and Vehrencamp, 1998; Fitzpatrick and Wellington, 1983; Wilson, 2000). A territory is defined as ‘an area occupied more or less exclusively by animals or groups of animals by means of repulsion through overt aggression or advertisement’ (Wilson, 2000). Most reported examples of territorial behaviour involve males defending resources related to reproduction. Territory holders usually advertise their space with chemicals or sounds to deter intruders, but if a resident is challenged, contests can escalate to physical aggression, sometimes resulting in death. Although these are the most typical territorial scenarios reported, many interesting exceptions are not well studied (Baker, 1983; Fitzpatrick and Wellington, 1983; Wilson, 2000). Here, we report on one of those exceptions – a caterpillar no larger than 2 mm, that vehemently defends the tip of a leaf against intruders using complex leaf-borne vibrations.

The warty birch caterpillar of the two-lined hooktip moth (*Falcaria bilineata*; Lepidoptera: Drepanidae) occurs across North America, feeding on birch (*Betula* spp.; Betulaceae) and alder (*Alnus* spp.; Betulaceae) leaves (Rose and Lindquist, 1997; Wagner, 2010). These caterpillars are known for their warty appearance and their arched, dragon-like resting stance (Mosher, 1917; Wagner, 2010), as well as their ability to produce vibrations (Bowen et al., 2008). The moths lay eggs in rows on twigs and leaves of host trees (Bowen et al., 2008; Dyar, 1894). Upon hatching, the tiny neonates (0.5 to 2 mm) wander on the leaf surface, where they are highly vulnerable owing to their lack of protective adaptations such as shelter building, group living or leaf mining (Bowen et al., 2008; Dyar, 1894; L.M.T., unpublished observations), which are characteristic of other neonate caterpillars (see Zalucki et al., 2002). A previous study reported anecdotally that early instar *F. bilineata* produced vibrations and tended to reside solitarily on leaves, suggesting that these larvae may be territorial (Bowen et al., 2008), although this hypothesis was not tested.

In this study, we tested two hypotheses. First, that neonate warty birch caterpillars are territorial. We predicted that: (i) caterpillars occupy an exclusive area on the leaf; (ii) undisturbed caterpillars on this space will occasionally advertise; (iii) when confronted with a conspecific, the resident usually retains the space (i.e. exhibits resource holding power); and (iv) sometimes intruders take over the space (Baker, 1983; Fitzpatrick and Wellington, 1983; Wilson, 2000). Second, that vibratory signalling functions in territorial contests. We predicted that: (i) caterpillars produce vibratory signals (i.e. vibrations distinct from those produced by crawling or chewing); (ii) signalling rates of an established resident escalate as an intruder approaches; (iii) residents signal more than intruders; and (iv) if an intruder takes over the territory, then it begins to signal (Bradbury and Vehrencamp, 1998; Wilson, 2000). These hypotheses were tested by assessing and characterizing territorial behaviours, and by staging encounters between established residents and conspecific intruders. Why these tiny caterpillars might defend leaf tips, and how complex vibrations function to fend off intruders, are discussed.

## MATERIALS AND METHODS

### Insects

Caterpillars of *Falcaria bilineata* (Packard 1864) were reared from eggs laid by wild-caught female moths collected at ultraviolet-emitting lights, including mercury vapour bulbs, 15 W ultraviolet collecting lights and LepiLED Maxi Switch LEDs (Brehm, 2017). Moths were collected from the Queen's University Biology Station (Chaffey's Lock, ON, Canada, 44.5788°N, 76.3195°W), and other regions near Ottawa, ON, Canada (45.4215°N, 75.6972°W) between May and June 2023, as well as from Howe Bay (Prince Edward Island, Canada, 46.302770°N, 62.408695°W) in August 2009. Moths laid eggs on different substrates (e.g. leaves, paper, twigs) in glass or screened enclosures, and upon hatching, neonate larvae were transferred to leaves on cuttings of paper birch (*Betula papyrifera*; Betulaceae) and maintained in jars or plastic bins until used in experiments. Only the first instars (1–3 days old) were used in experiments.

### Choosing a leaf location

**Fig. 1.**
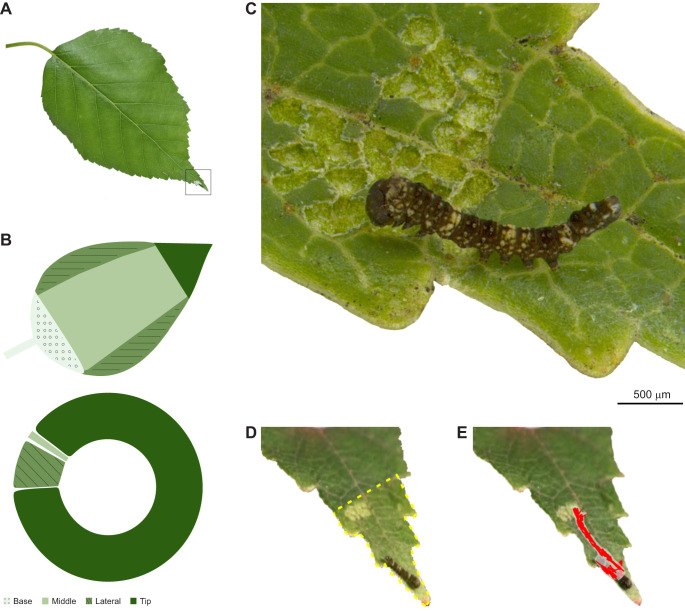
**Leaf tip territories of neonate warty birch (*Falcaria bilineata*) caterpillars.** (A) Leaf of paper birch (*Betula papyrifera*) showing the territory location (indicated by box). (B) Choices of territory location. Top panel shows leaf regions. Bottom panel shows where larvae established their territories, primarily on the leaf tip. (C) Neonate larva on its territory, showing the feeding scar. Scale bar, 500 µm. (D) Leaf tip territory of a neonate caterpillar, showing the territory border in yellow. (E) Tracked movements of caterpillar between the feeding spot and resting area over a 30-min period, shown in red.

We first assessed whether larvae show a preference for specific leaf locations. To do this, a total of 59 replicates were conducted. Each replicate involved a single birch leaf (6–9 cm long×4–6 cm wide) on a birch twig inserted into a hole in the lid of a water-filled plastic vial. Each vial was then enclosed within a plastic container lined with moistened paper towels. One neonate larva (<24 h after hatching) was carefully transferred using a fine-tipped paintbrush to the centre and adaxial side of the leaf. After an interval of 12–24 h, leaves were inspected to document where the larva was established, as indicated by the presence of a feeding scar and a silk mat. Leaf location preferences were scored as one of four locations (base, middle, lateral or tip) and side of the leaf (abaxial or adaxial) (see Fig. 1). Preference for leaf location was evaluated using a chi-square test, which ascertained whether the distribution of established locations significantly deviated from the expected occurrence of 25%.

### Territorial behaviour

Vibroacoustic and video recordings were conducted on established individual residents to describe and delineate the home range area, and to characterize how they behaved in this space before and after introducing a conspecific intruder to the leaf. These recordings were also used to identify and characterize vibratory signals and events. A total of 40 videos were analyzed, and subsets of these videos were used to address different questions. How videos and corresponding .wav files were sampled and analyzed to address different questions are reported below.

### Recording setup

Simultaneous video and vibration recordings were conducted to study larval territorial behaviours. A plastic vial with a twig containing a single birch leaf with one established larva (as described above) was clamped to a sturdy stand positioned on an antivibration table. Vibratory signals were recorded from the leaf surface using a laser Doppler vibrometer (PVD-100, Polytec Inc., Waldbronn, Baden-Württemberg, Germany) (velocity 20 mm s^−1^; high pass filter off; low pass filter 20 kHz) with the laser beam focused on reflective tape (5.5 mm diameter) affixed to the upper (adaxial) leaf surface 2–3 cm from the established caterpillar. Forty trials were videotaped using a camcorder (Canon XA11 or Canon Vixia HF G20, or Sony HDR-XR520V Handycam) equipped with a macro close-up lens (Raynox DCR-250 or DCR-100, Yoshida Industry Co., Tokyo, Japan) and coupled with the laser vibrometer analogue output connected to the microphone port. In a subset of trials, the laser analogue was also connected to a data recorder (Marantz PMD 671, Marantz Corp., Kanagawa, Japan) set to a sampling rate of 48 kHz. All trials were performed within an acoustic chamber (C-14A MR, Eckel Industries Ltd, Cambridge, MA, USA).

### Vibratory signals

Vibratory signals were identified and characterized in two stages. First, close-up videos (+laser vibrometry) of first instar larvae in different behavioural contexts (e.g. undisturbed or in the presence of conspecific; see Results) were watched at reduced playback speed, while listening with headphones, to correlate vibrations with body movements. Vibrations were designated as ‘signals’ if they were generated by specialized body movements (i.e. did not occur incidentally because of activities such as chewing or crawling) and had a high signal-to-noise ratio. In total, ∼29 h of video footage was reviewed. Once vibrations were categorized as signals, each signal type was analyzed for temporal and spectral characteristics based on a subset of eight audio files in .wav format (∼7 h of recordings) from different undisturbed individuals. Initial temporal analysis indicated that signals are often produced in bouts, defined as a series of signals separated by at least 1 s of inactivity. Four temporal characteristics were measured altogether: (i) bout duration, (ii) total number of signals within a bout, (iii) number of each signal type within a bout and (iv) signal duration. The peak frequency was obtained for each signal after applying a FFT (sampling frequency: 48,000; window type: Hanning; window size: 1024; overlap: 50). Data were collected by selecting between three and six bouts from eight individuals, resulting in a total of 34 bouts, and 212 different signals (162 buzz scrapes and 50 drums). Acoustic analyses were performed using the software Audacity^®^ (version 3.6.1; https://audacityteam.org/).

### Territory boundaries and movement patterns

Video recordings of established larvae were reviewed to describe the boundaries of the home range (an area that an animal habitually patrols), which is used as a proxy for territory (see Wilson, 2000), and activity patterns within this space. We tracked an individual's movements frame-by-frame using idtracker.ai (version 5.2.11) (Romero-Ferrero et al., 2019) running within the Anaconda Software Platform (version 2-2.4.0; https://anaconda.com), which provides the necessary environment for the tool. A total of 34 videos of individuals were used for home range measurements. However, only videos with an identification accuracy greater than 95% were included in the tracking analysis to ensure reliable tracking (*n*=18 videos). From the selected videos, we identified and measured the core area used by the caterpillar where they fed, rested, signalled and laid silk. Measurements were made using CorelDraw 2021 (Corel, Ottawa, ON, Canada). The trajectory obtained from the videos was imported into R software (version 4.3.3; https://www.R-project.org/) running in the RStudio interface (version 2023.12.1+402; http://www.rstudio.com/). We generated the caterpillar pathways using the geom_path function from the ggplot2 package, using a screenshot from the video as a background layer. All video analysis procedures were performed on a computer running Windows 11 Pro 64-bit with an Intel^®^ Core i7-9750H CPU (2.60 GHz×12), 32 GB RAM and an NVIDIA^®^ GeForce^®^ GTX 1660 Ti Max-Q GPU.

### Territorial encounters

To assess territorial behaviour, 18 encounters were staged between a resident and a conspecific intruder. For each trial, an established first instar larva (the resident) was set up in the recording apparatus (see ‘Recording setup’, above) and recorded undisturbed for 30 min. Then, a conspecific first instar larva (the intruder) randomly selected from the rearing container was carefully placed, using a fine-tipped paintbrush, 3–5 cm from the resident on its territory. Both larvae were then recorded for an additional 30 min. Video recordings were reviewed at reduced playback speed to mark various behaviours including signalling events and locations of residents and intruders relative to the territory. A ‘win’ was defined as the individual occupying the territory at the conclusion of the trial. Time sequenced files were created using JWatcher Video (version 1.0; https://www.jwatcher.ucla.edu/), and data files from JWatcher were exported to Microsoft Excel. To test whether the signalling rates of a resident escalate as an intruder approaches, we assessed the types and rates of signalling by the contestants across three contexts: (i) undisturbed resident (the resident during the 30 min prior to adding the intruder), (ii) intruder on leaf but outside territory (when the intruder was on the leaf but not within the boundary of the resident's territory) and (iii) intruder on resident's territory (when the intruder had crossed the territory boundary of the resident). Signalling rates were calculated for each trial by dividing the total number of vibratory signals (buzzes+drums) by the total recording duration in each trial. This calculation was performed for each individual (resident or intruder) within each context (undisturbed, *n*=18; intruder on a leaf, *n*=13; or intruder in resident territory, *n*=8). The resident signalling rates were analyzed using a generalized linear model (GLM) with deviance analysis, incorporating sample-size-based weights to account for the differing number of replicates across contexts. Residual normality and homoscedasticity were assessed using the DHARMa package. To compare whether residents signal more than intruders, we used the paired *t*-test to evaluate signalling rates between residents and intruders within the same context. Finally, we assessed resource holding power by scoring the number of wins for each individual (‘resident’ and ‘intruder’). To evaluate the relationship between individual identity and win probability, we fitted a binomial GLM. Model diagnostics were checked using the DHARMa package. All analyses were conducted in R software (version 4.3.3; https://www.R-project.org/) within the RStudio interface (version 2023.12.1+402; http://www.rstudio.com/).

## RESULTS

### Territory location and layout

Caterpillars showed a strong preference to settle on the leaf tip (Fig. 1). The probability of establishing on any of the four scored leaf regions is different than expected by chance (χ^2^=126.83, d.f.=3, *P*<0.0001), with the highest preference for the leaf tip (88.14%, *n*=52/59), then the lateral edges (10.17%, *n*=6/59) and the middle (1.69%, *n*=1/59). No individuals were established at the leaf base (Fig. 1). Also, the majority (93.22%, *n*=55/59) were established on the adaxial surface of the leaf (χ^2^=44.085, d.f.=1, *P*<0.0001).

**Fig. 2.**
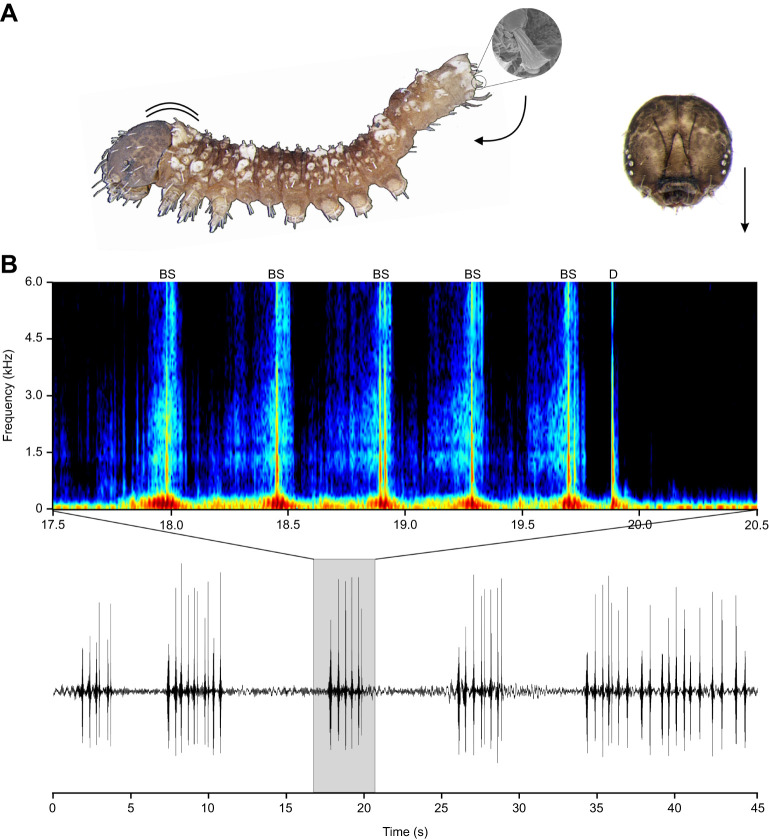
**Vibratory signals produced by neonate *Falcaria bilineata* caterpillars.** (A) Body movements associated with buzz scrape (BS) and drum (D). For buzz scrapes, the caterpillar begins in resting position, with only the prolegs in contact with the leaf. The vibration begins with a tremulation of the anterior body, while the anal segment with anal ‘oar’ structures (shown in inset) is lowered and dragged anteriorly across the leaf surface. For drums, the anterior body (head+thorax) rapidly strikes the leaf surface. (B) Five signalling bouts are shown in the waveform of the lower panel. Each bout comprises a succession of buzz scrapes and drum signals. The spectrogram (upper panel) representing the third bout, shows six signals (five buzz scrapes followed by a single drum).

**Fig. 3.**
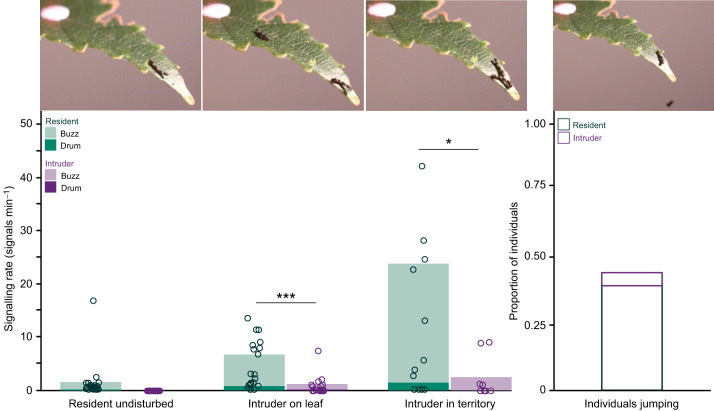
**Territorial behaviour of neonate *Falcaria bilineata* caterpillars, showing vibratory signalling rates across three contexts.** (1) Resident undisturbed (*n*=18), (2) intruder on leaf (but outside of the resident's territory) (*n*=13) and (3) intruder in territory (*n*=8). The proportion of jumping events (*n*=18) is also shown. Signal rates of the resident escalate significantly between contexts as the intruder is introduced to the leaf and enters the resident's territory. Intruders signal significantly less than do residents in both ‘on leaf’ and ‘in territory’ contexts. Filled bars represent the mean vibratory signalling rates of residents (green) and intruders (purple) for each context and signal type. Individual data points represent the signalling rate for each trial. Non-filled bar indicates the proportion of individuals jumping. A GLM with sample-size-based weights was used to test the resident signalling rates across contexts. A paired *t*-test was used to compare signalling rates between residents and intruders within each context (*P<0.05; ***P<0.001).

Territories of established residents on leaf tips were defined based on movement patterns. Resident movements were tracked for 18 individuals over a total time period of 8 h (±28 min per video). The core area of use (8.02±1.25 mm^2^) (*n*=18) is where larvae typically eat, rest and signal (Fig. 1; Movie 1). Notably, undisturbed residents did not wander on the leaf beyond the feeding scar, and based on this, territory boundaries were delineated as extending from the leaf tip to the proximal edge of the feeding scar (Fig. 1; Movie 1). The feeding scar, resulting from leaf skeletonization, is located at a distance of 11.56±0.70 mm (*n*=34) from the distal edge of the leaf tip. This area from the feeding scar to the leaf tip as well as the leaf edges is covered by a silk mat, with no shelter. The very tip of the leaf was never consumed and remained green (*n*=35/35) (Figs 1–3; Movies 1 and 3).

### Vibratory signals

First instar caterpillars produce two distinct vibratory signals: drums and buzz scrapes (Fig. 2; Movie 2). Drums are generated when the anterior body rapidly strikes the leaf surface (Fig. 2). It was not always possible to confirm which part(s) of the body (head, thorax or both) struck the leaf, as the movement sequence could not be resolved with our video frame rate (29.97 frames s^−1^) (Movie 2). Drums can occur on their own, but most often occur in combination with buzz scrapes (Fig. 2; Movie 2). Buzz scrapes are multicomponent signals involving simultaneous body tremulation, stridulation, and often percussion (from the inclusion of the drums during the buzz scrapes) (Fig. 2; Movie 2). A buzz scrape begins with the caterpillar head+thorax and terminal anal segments (A7–A10) elevated off the leaf surface (Fig. 2; Movie 2). The vibration begins with a low amplitude, vertical tremulation of the head+thorax accompanied by the anal segment being lowered and dragged anteriorly across the leaf surface. This type of stridulation involves a pair of oar-shaped setae (posterior proctal, PP1) on the terminal abdominal segment that are specialized for generating vibrations in some Drepanoidea (Scott et al., 2010a,b; Yack et al., 2001) (Fig. 2). At the end of the buzz scrapes, the head and anal segments are raised to return to the resting posture. Although the buzz scrape can occur on its own, one or more drums often occur simultaneously during the buzz scrapes. Vibratory signals are often produced in bouts, comprising a rapid succession of buzz scrapes and terminating with one or more isolated drums (Fig. 2; Movie 2).

Data on signal characteristics were collected using a total of 34 bouts, and 212 different signals (162 buzz scrapes and 50 drums) produced by eight individuals. Drum duration was 48.64±1.21 ms. Drums were broadband with three prominent peaks at 188.96±13.14, 572.0±22.52 and 1321.77±52.21 Hz. Buzz scrapes were 243.51±6.82 ms in duration and broadband with three prominent peaks at 139.10±3.02, 489.41±13.01 and 1011.05±26.25 Hz (Fig. 2; Movie 2)*.* The duration of signalling bouts was 2.22±0.25 s. Bouts contained an average of 5.59±0.40 signals (Fig. 2; Movie 2).

Other vibrations recorded during our experiments included a low amplitude vertical tremulation of the anterior body that may occur on its own or preceding a buzz scrape, as well as vibrations incidental to chewing and crawling. These did not meet the criteria for being ‘signals’ and were not characterized or scored.

### Territorial contests

Eighteen encounter trials were conducted to assess the territorial behaviour *F. bilineata* caterpillars. During the 30 min prior to introducing an intruder, undisturbed residents spent most of their time feeding or resting, and occasionally signalling at a mean rate of 1.64±0.97 signals min^−1^ (*n*=18) (Fig. 3). When an intruder was added to the leaf (but was not on the territory) the resident typically stopped feeding or laying silk, moved to the green part of the leaf tip, and increased signalling (6.92±1.47 signals min^−1^ (*n*=13) (Fig. 3; Movie 3). Overall, resident signalling rates escalated as an intruder approached (*F*_2,36_=15.272, *P*<0.001). When an intruder entered a resident's territory (in 8/18 trials), resident signal rates increased to 24.41±7.05 signals min^−1^ (*n*=8) (Fig. 3; Movie 3). Intruders also signalled when on the leaf (1.21±0.69 signals min^−1^, *n*=13), and in those individuals that crossed into the resident's territory, their signal rates increased to 2.55±1.42 signals min^−1^ (*n*=8) (Fig. 3). Overall, residents signalled significantly more than intruders, both when the intruder was on the leaf (but outside the territory) (∼5.7 times more, *t*=3.86, d.f.=12, *P*=0.002) and when the intruder was on the resident's territory (9.5 times more, *t*=2.83, d.f.=7, *P*=0.025). No biting or pushing by either the resident or intruder occurred during these encounters. However, residents frequently jumped off the leaf tip and hung on a silk thread, a behaviour known as ‘lifelining’ (Brackenbury, 1996) (Fig. 3, Movie 3). Jumping occurred in 44.4% of all trials (*n*=8/18 trials), and always when the intruder approached the resident and made body contact. Residents never jumped off the leaf when they were alone. When resident jumping occurred, the intruder either remained on the territory and began to feed (3/8 trials) or wandered away (5/8 trials).

The outcome of each contest (i.e. who ‘won’ the trial) was assessed based on which contestant remained on the territory at the end of the trial (Fig. 3; Movie 3). In 14 out of 18 trials, one of the contestants occupied the territory at the end of the contest, whereas in 4 out of 18 trials, neither the resident nor the intruder remained on the territory, and this was due to the resident hanging on a thread while the intruder had walked away at the end of the 30 min recording period. Among the 14 contests where there was a clear ‘winner’, residents secured the territory in 71.42% of contests (*n*=10/14), whereas intruders won 28.58% of contests (*n*=4/14). Notably, none of the trials (0/18 trials) ended with both individuals on the territory.

## DISCUSSION

Some of the most elaborate behaviours in the animal kingdom occur during territorial exchanges (Wilson, 2000). Here, we show that tiny caterpillars, no longer than 2 mm, produce complex multicomponent vibratory displays while defending a leaf tip. We discuss why neonate caterpillars would guard a leaf tip, and how complex vibratory signalling might deter intruders.

The first hypothesis tested in this study was that neonate warty birch caterpillars are territorial. Key predictions for animal territoriality are that individuals occupy an exclusive area, that they defend this area through advertisement and/or physical aggression, and that usually, the resident wins conflicts, exhibiting resource holding potential (Baker, 1983; Fitzpatrick and Wellington, 1983; Wilson, 2000). Our results align with these predictions. Neonate warty birch caterpillars show a strong preference to establish themselves at the distal tips of birch leaves. They occupy this space exclusively, where they lay a silk mat, rest, feed and occasionally advertise by signalling. When confronted with an intruding conspecific, the resident usually retained the territory (*n*=10/14), but occasionally the intruder occupied the territory at the end of the trial (*n*=4/14). No trials ended with two individuals settling on the territory. These results provide compelling evidence for territorial behaviour against conspecific intruders in neonate caterpillars, and to the best of our knowledge, this is the first documented instance of an insect defending a leaf tip.

Why defend a leaf tip? A hallmark of animal territoriality is to gain access to valuable resources that are in short supply, such as food (Wilson, 2000). Although leaves on a birch tree are not a limited resource, the leaf tips in particular may provide nutritive benefits such as being more tender (e.g. Malishev and Sanson, 2015) or having a lower density of trichomes. Such traits would be beneficial to neonate caterpillars that can experience high mortality rates due to plant defences (Zalucki et al., 2002). At present there is no evidence that birch leaf tips are more tender or have fewer trichomes than other parts of the leaf, but this would be interesting to explore. An alternative hypothesis to explain the selective advantage of residing on a leaf tip is that this location could facilitate escape from predators. Early instar of *F*. *bilineata* have few antipredator defences that are commonly employed by other neonate caterpillars (see Zalucki et al., 2002). They live solitarily on the leaf surface and lack a protective shelter or other apparent physical defences (Bowen et al., 2008). Our results show that upon being contacted by an intruding conspecific, a significant proportion of residents jumped off the leaf tip, yet remained attached to the leaf by a silk thread. This behaviour, described as lifeline hanging, occurs when some caterpillar species become alarmed (Brackenbury, 1996). Most species that employ lifeline hanging are free-living (Sugiura and Yamazaki, 2006) and may use this escape mechanism as an alternative to building a protective silk shelter. We propose that for *F*. *bilineata* neonates, the leaf tip facilitates predator avoidance for a couple of reasons. First, residing on the leaf tip would allow residents to predict the direction of a crawling predator such as a spider, lacewing or beetle, which would approach the leaf from the petiole. Second, leaf tips have been shown to have higher vibration amplitudes when compared with other parts of the leaf (Casas et al., 1998; Meyhöfer et al., 1994), which might facilitate escape. The flexibility of the leaf tip could act as a springboard for jumping, or could amplify the vibrations resulting from the crawling movements of an approaching predator, allowing for early detection. Another possible advantage of residing on a flexible leaf tip is to enhance the amplitude of the resident's signal to make the resident appear larger or more dangerous (Wilson, 2000) (see also discussion of this point below). There are few examples of animal territories that function primarily as escape routes (Baker, 1983; Fitzpatrick and Wellington, 1983; Wilson, 2000), but we argue that this is a likely explanation for territoriality in neonate *F. bilineata*. To further test how residing on a leaf tip enhances survival, future experiments could assess the biomechanics of birch leaves, including the spatial distribution and transmission properties of vibrations, as well as the survival benefits of residing on the leaf tip in the presence of natural predators.

The second hypothesis tested in this study was that vibratory signals function in territorial defence. The functions of animal territorial signals are to advertise the presence of an owner, define territorial boundaries and repel intruders (Bradbury and Vehrencamp, 1998). Our results support several key predictions for this hypothesis. First, undisturbed residents occasionally advertise by stopping feeding or laying silk, often moving to the very tip of the leaf, and producing short bouts of signalling. Second, when a conspecific neonate was placed on the leaf, the resident increased its rate of signalling, and these rates escalated further when the intruder crossed the territory boundary. Third, residents signal more than intruders in all contexts. Interestingly, territorial signalling in *F. bilineata* neonates differs from other examples in insects. For example, in most reported insect territorial systems, signalling often communicates the ability to fight, with signal escalation resulting in physical aggression (Baker, 1983). However, our results show that signal escalation in *F. bilineata* never results in physical fighting such as biting, pushing or thrashing. Instead, contests appear to be resolved by vibratory signalling. This behaviour could reflect a survival strategy for soft-bodied larvae, where biting could be lethal. Such non-aggressive territorial interactions might be more widespread among larvae or other non-weaponized insects, yet have been underreported in the literature. Moreover, vibratory signalling in the context of territorial conflicts is not well documented in insects in general (e.g. Turchen et al., 2022; Virant-Doberlet et al., 2023), with most territorial insects reported to use visual, chemical or airborne-sound displays (Baker, 1983; Fitzpatrick and Wellington, 1983). For small, flightless insects such as caterpillars that are substrate-bound and lack good vision, vibration-mediated signals may be the optimal communication modality. In fact, there is a growing number of examples of larger, late instar (IV,V) caterpillars defending whole leaves or silk shelters and sometimes incorporating vibratory signalling [e.g. *Drepana arcuata* (Drepanidae) (Yack et al., 2001), *Falcaria bilineata* (Drepanidae) (Bowen et al., 2008), *Oreta rosea* (Drepanidae) (Scott et al., 2010a,b), *Tethea or* (Drepanidae) (Scott and Yack, 2012) and *Caloptilia serotinella* (Gracillariidae) (Fletcher et al., 2006)]. Overall, our results provide strong support that territorial defence is mediated by complex vibratory signalling even in tiny hatchlings. We propose that this form of territorial signalling may be more common in substrate-bound insects than currently reported, but overlooked by researchers owing to the lack of awareness and capability of recording these vibrations.

Recent studies have significantly enhanced our understanding of vibrational communication, particularly in environments where multiple individuals – both conspecific and heterospecific – can detect emitted signals (Virant-Doberlet et al., 2023). To explain how vibratory territorial behaviour evolved in *F. bilineata*, it is important to consider their natural habitat. Adult *F. bilineata* females lay eggs in rows on leaves (Bowen et al., 2008). Although eggs laid in clusters can be a predictor of sociality in Lepidoptera larvae (Costa, 2006; Despland, 2021), warty birch caterpillars live solitarily throughout their development (Bowen et al., 2008), and therefore egg clustering may function for some other purpose, such as to prevent desiccation (Despland, 2021). Upon hatching, neonates, which experience high mortality owing to a number of biotic and abiotic factors, are under pressure to disperse immediately and to establish themselves and begin feeding (see Zalucki et al., 2002). In animals that start from the same place prior to dispersal, the search pattern for resources often overlaps between individuals (Krause and Ruxton, 2002). In neonate *F. bilineata*, emerging larvae searching for leaf tips are likely to have frequent encounters with conspecifics, and thus competition for leaf tips may drive territorial behaviour. Once established on a leaf tip territory, intermittent signalling bouts by undisturbed residents may function to broadcast a message of leaf-tip occupancy to wandering conspecifics. One of the most direct forms of territorial maintenance is through repetitious signalling, directed not at a specific individual, but broadcast into the environment (Wilson, 2000). Thus, signalling could be a species-specific message announcing, ‘This leaf tip is already occupied’. A group of neighbours on a tree branch may maintain spacing by forming a communication network by signalling on a regular basis to advertise occupancy, distance and possibly, identity (Bradbury and Vehrencamp, 1998; McGregor, 1993). An alternative but not mutually exclusive hypothesis is that signalling by territory holders may extend to the broader community on the tree, including potential predators or other competitors for leaf space (e.g. Cappuccino, 1993; Fitzpatrick and Wellington, 1983; Rose and Lindquist, 1997). For example, complex vibratory signals may function as deimatic displays (Low et al., 2021), or by mimicking something more dangerous, such as the vibratory territorial or mating signals of a jumping spider (e.g. Elias et al., 2003, 2008). Interestingly, such spider vibratory displays can also be multicomponent and described as having ‘buzzes’, ‘thumps’ and ‘scrapes’ (Brandt et al., 2020; Elias et al., 2003).

To better understand why these tiny caterpillars defend leaf tips using complex vibrations, future research must consider biotic and abiotic factors in their natural habitat, including vibrations produced by conspecifics, predators and other competitors for leaf space, as well as noise from wind and rain (e.g. Guedes et al., 2012; Turchen et al., 2023). For example, observing how recently hatched neonates distribute themselves on a host plant would inform us about the role of vibrations in conspecific spacing. Also, identifying heterospecifics that occur on the host plant, including predators, parasitoids and competitors, would provide further insights into other possible functions of signalling, and whether the caterpillars can discriminate between different species. Finally, examining plant traits that influence vibration propagation (e.g. Casas et al., 2007; Magal et al., 2000; Velilla et al., 2020) could reveal how different regions of a leaf resonate with these signals and the distances they can travel. Such information can help to better understand caterpillar territoriality, spacing behaviours and other selection pressures that drive their interactions and communication in the natural vibroscape.

## Supplementary Material

10.1242/jeb.249796_sup1 Supplementary information
