# Supplementary material for: Buzzing boundaries: tiny caterpillars vibrate to defend leaf tip territories
Source: J Exp Biol. 2025 Apr 1;228(7):jeb249796. doi: 10.1242/jeb.249796 (PMC11993261; doi:10.1242/jeb.249796)
Supplement: Supplementary information [file jexbio-228-249796-s1.pdf]

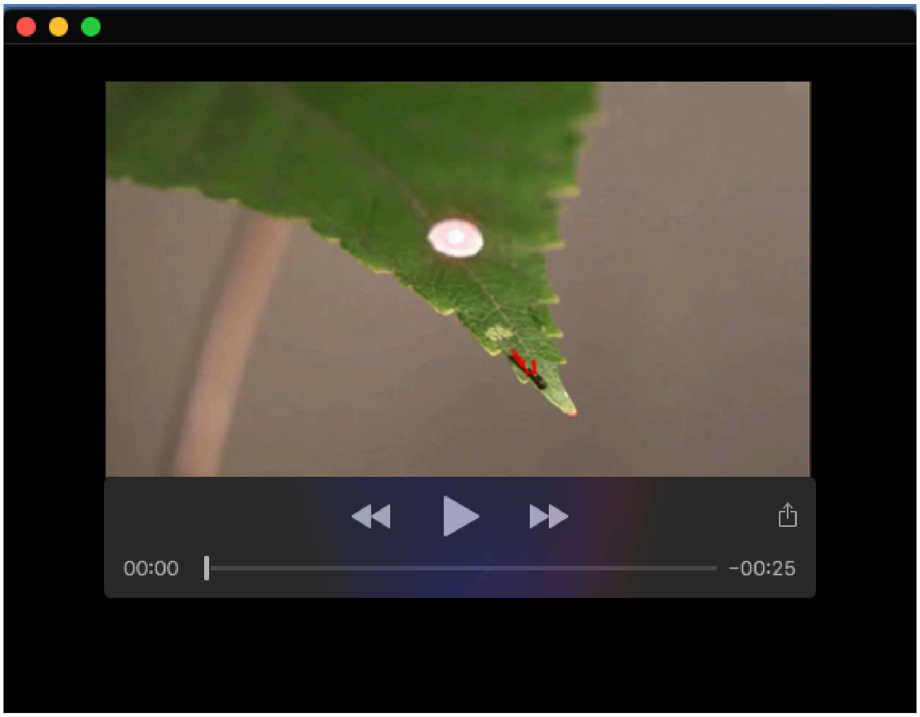

Movie 1. An undisturbed neonate *F. bilineata* caterpillar occupies its territory on the tip of a birch tree leaf, moving between the feeding scar and the green part of the leaf tip. Movements are tracked over a period of 30 minutes.

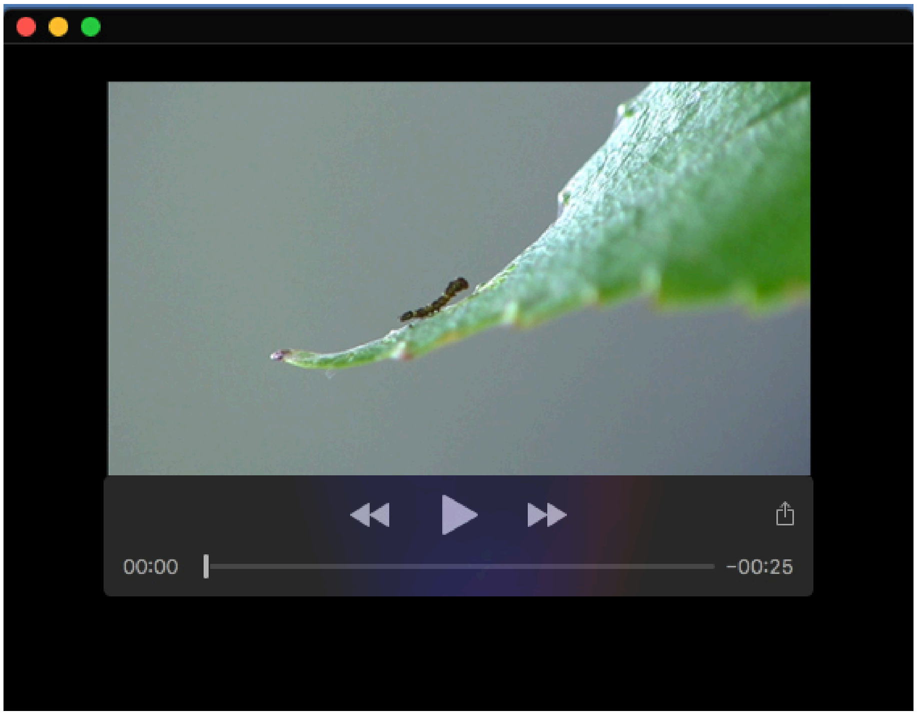

Movie 2. Vibratory signalling in neonate *F. bilineata* caterpillars. The first part shows body movements associated with vibratory signalling. The second part shows four signalling bouts, with the waveform at top, and the spectrogram at the bottom.

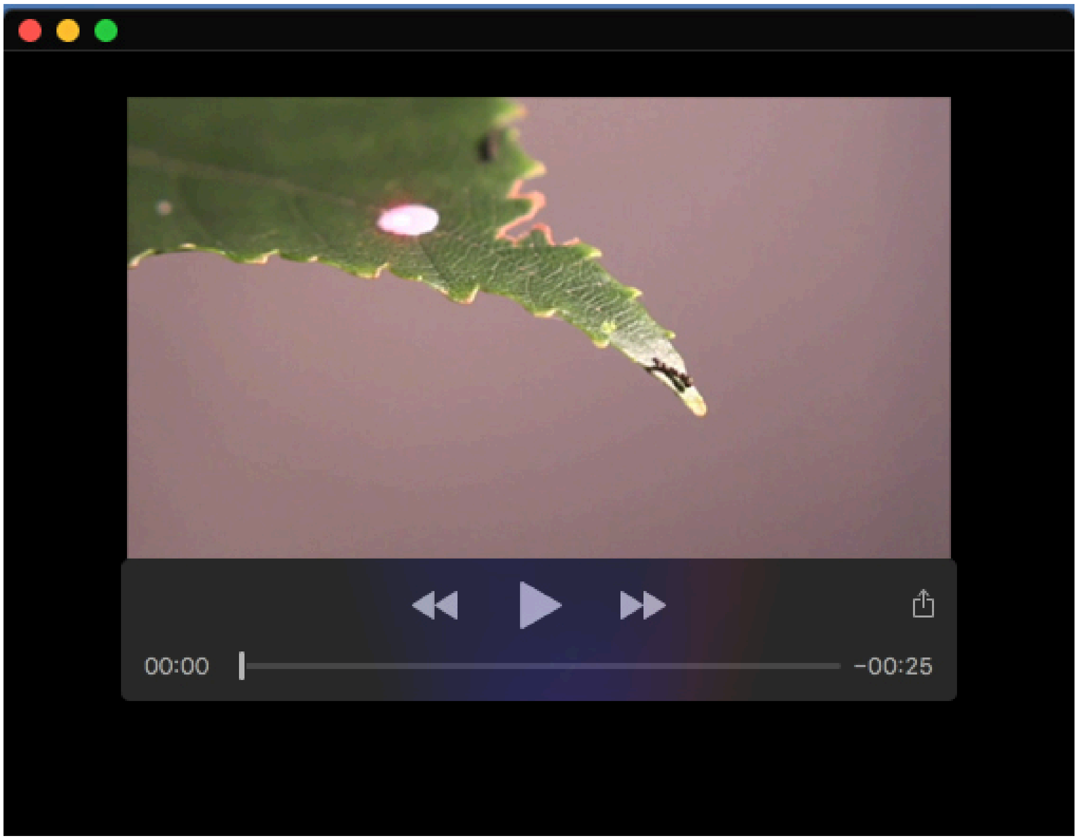

Movie 3. Territorial encounters between neonate *F. bilineata* caterpillars. The first video shows a resident being approached by a conspecific intruder. The resident begins signalling and jumps off the leaf tip on a silk line when the intruder enters the territory and contacts the resident. The intruder proceeds to the feeding scar and begins feeding. Note that the 'wobble' sound in the background is noise from the laser vibrometer. The second part shows a resident resting being approached by a conspecific intruder and in this case the resident signals and the intruder leaves the territory.
